# Are exceptions justified in the current heart allocation system?

**DOI:** 10.1016/j.jhlto.2023.100014

**Published:** 2023-11-02

**Authors:** Maarten Coemans, Amrusha Musunuru, Les James, Deane Smith, Nader Moazami, Dorry Segev, Sommer Gentry

**Affiliations:** a Department of Microbiology, Immunology and Transplantation, KU Leuven, Leuven, Belgium; b Leuven Biostatistics and Statistical Bioinformatics Centre (L-Biostat), KU Leuven, Leuven, Belgium; c Department of Surgery, NYU Grossman School of Medicine, New York, New York; d Department of Cardiothoracic Surgery, NYU Grossman School of Medicine, New York, New York; e Department of Population Health, NYU Grossman School of Medicine, New York, New York

**Keywords:** heart allocation, heart transplant, waitlist mortality, exceptions

## Abstract

**Background:**

In the current 6-tiered heart allocation system, waitlisted candidates frequently receive an exception to any of the top 4 allocation statuses. Studies have shown varying results regarding the mortality risk of exceptions compared to standard criteria listings in the top 2 statuses. It remains unclear whether exception statuses in the current allocation system are justified.

**Methods:**

We performed a retrospective cohort study on all adults waitlisted for heart transplant between November 1, 2018, and December 31, 2022, contained in the United Network for Organ Sharing database. The waitlist mortality and transplantation rate of all exception status and standard criteria listings were compared and ranked, using status 6 as the reference status, in univariable and multivariable Cox models including status as a time-dependent variable.

**Results:**

A total of 17,116 heart waitlist candidates were included in the study. The waitlist mortality rates of exceptions in status 1 and 2 were lower than, but close to, the mortality rates of their standard criteria counterparts: status 1 standard (hazard ratio) HR 43.47 (*p* < 0.001), status 1 exception HR 31.48 (*p* < 0.001), status 2 standard HR 10.21 (*p* < 0.001), status 2 exception HR 7.76 (*p* < 0.001). The mortality rate of exceptions in status 3 and 4 was higher than, but also close to, the mortality rate of their standard criteria counterparts: status 3 standard HR 2.61 (*p* < 0.001), status 3 exception HR 4.18 (*p* < 0.001), status 4 standard HR 1.31 (*p* = 0.104), and status 4 exception HR 2.13 (*p* < 0.001).

**Conclusions:**

Based on waitlist mortality over a longer study period than previous analyses, high-priority exceptions are appropriately placed in the current heart allocation system. The current allocation system requires a large fraction of candidates to seek exceptions, suggesting that we should focus on objective data to risk stratify all heart waitlist candidates, and thereby reduce the need for exceptions.

Following the implementation of the 6-tier heart allocation system on October 18, 2018, there were more than 20,000 heart transplants performed in the US.^1^ There are 3411 candidates on the heart waitlist, with 873 new additions in 2023.^1^ One of the main goals of the 6-tier allocation system was to reduce exceptions compared to the previous 3-tier heart allocation system.^2^ However, the number of exception requests on the heart waitlist increased dramatically from 3.4% in the prepolicy period (2015-2018) to 17.8% in the postpolicy (2018-2021) period.^3^ Additionally, the proportion of recipients with an exception at the time of transplant increased from 10% to 32%.^3^

In the current allocation system, a heart waitlist candidate can receive status 1, 2, 3, or 4 by qualifying for an exception if the “transplant physician believes, using acceptable medical criteria, that a heart candidate has an urgency and potential for benefit comparable to that of other candidates at the requested status.”^2^ To understand the appropriateness of exception requests, several studies have compared the waitlist mortality between exceptions and standard criteria listings.^3^, ^4^, ^5^, ^6^ One early study reported exception listings had an increased rate of transplantation with no increased risk of waitlist mortality, when considering exceptions at every status as an aggregate group.^6^ Other studies yielded varying conclusions when comparing waitlist mortality between exceptions and standard criteria stratified by status and accounting for candidates’ status changes over time. One study reported a significantly lower waitlist mortality rate for status 1 and 2 exceptions compared to their standard criteria counterparts,^5^ while another study reported a significantly lower waitlist mortality rate for status 1 exceptions and no difference in waitlist mortality rate for status 2 exceptions compared to their standard criteria counterparts.^3^ These studies had a short inclusion period, from October 18, 2018, until the end of 2021, and differed in their definition of mortality events.

The Final Rule states that “*candidate priority rankings shall be ordered from most to least medically urgent*,” but the medical urgency of exception listings in the current heart allocation system remains unclear. We aim to clarify this by analyzing a longer study period, leveraging more data, and ranking the hazard of death for candidates listed at each status under exception and standard criteria.

## Materials and methods

### Patient population

Data were from the United Network for Organ Sharing (OPTN) March 2023 Standard Transplant, Analysis, and Research file. Our cohort consisted of all adult patients waitlisted for a heart transplant between November 1, 2018, and December 31, 2022. For every patient, the last waitlist status (1-6) on each day was retained and used to define the first active status, as well as subsequent status changes. Patients who were in inactive status for any length of time were retained in their prior status for our analysis. Demographics at listing were reported overall and stratified by first active status 1 to 4 (standard criteria and exception listing). Included demographic variables were sex, age, weight, body mass index (BMI), ethnicity,^7^ blood type, diabetes status, history of smoking, serum creatinine, functional status, inotrope use, extracorporeal membrane oxygenation (ECMO), invasive mechanical ventilation, listed for heart-kidney transplantation, cause of heart failure, cerebrovascular accident (CVA), stroke, automatic implantable cardioverter defibrillator, and mechanical circulatory support device (MCSD). Unless specified otherwise, our cohort had complete data on all of these variables. At the time of first active status, we retrieved the risk stratification variables from the status justification forms for status 1 to 4. The risk stratification variables were history of peripheral thromboembolic events, antiarrhythmic use, dialysis, vasoactive support, invasive mechanical ventilation, number of hospital admissions in the last year, number of prior sternotomies, cardiac index, cardiac output, central venous pressure, systolic blood pressure, diastolic blood pressure, left ventricular end diastolic pressure, mean pulmonary artery pressure, pulmonary artery diastolic pressure, pulmonary artery systolic pressure, pulmonary capillary wedge pressure (PCWP), mixed venous oxygen saturation, serum albumin, arterial lactate, aspartate aminotransferase, serum bilirubin, B-type natriuretic peptide (BNP), serum creatinine, international normalized ratio, serum sodium, blood urea nitrogen, Calculated Panel Reactive Antibody, and inotrope use. We reported the data availability of each of these risk stratification variables. Our primary end point, waitlist mortality, was a composite of OPTN removal codes 5 (“medically unsuitable”), 8 (“died”), and 13 (“candidate condition deteriorated, too sick for transplant”). The secondary end point was cardiac transplantation (removal codes 4, 14, 15, 18, 19, 21, 22).

The data reported here have been supplied by the UNOS as the contractor for the Organ Procurement and Transplantation Network. The interpretation and reporting of these data are the responsibility of the author(s) and in no way should be seen as an official policy of or interpretation by the OPTN or the U.S. Government. This study was deemed exempt from ongoing oversight by the NYU Langone Institutional Review Board and complies with the International Society for Heart and Lung Transplantation ethics statement.

### Statistical analysis

Continuous variables were reported as means and variances, or medians and interquartile ranges where appropriate. Categorical variables were summarized via frequencies and percentages. For patients reaching a status via an exception, we created a Sankey plot that showed these patients’ first active status. All consequent time-to-event analyses started at the day of first active status and ended at the day of candidate removal (see earlier codes), or at March 31, 2023 (administrative censoring date). Waitlist mortality was censored for transplantation, and vice versa. Extended Kaplan-Meier curves^8^ were drawn in order to visually compare the waitlist mortality rate and transplantation rate between standard criteria listing and exception listing in status 1 to 4. Extended Kaplan-Meier curves differ from standard Kaplan-Meier curves in that they allow patients to change status (risk set) over time. In a univariable Cox model, status was also included as a time-dependent covariate, yielding instantaneous hazard ratios of all statuses compared to status 6 (= reference group). We additionally calculated the hazard ratios for waitlist mortality and transplantation of exception listing vs standard criteria listing (contrasts) within statuses 1 to 4. In multivariable Cox models, we adjusted for sex, race, blood type, diabetes status, smoking history, age, BMI, and estimated glomerular filtration rate.

SAS 9.4 (SAS Institute, Cary, NC) was used for all statistical analyses. *p*-values were based on 2-sided hypothesis tests and were considered significant when below 0.05.

## Results

### Demographics

In total, 17,116 heart waitlisted patients were included in the study, with a median follow-up of 50 days (interquartile range (IQR), 13-229). Among candidates listed by standard criteria, first active status 1, 2, 3, 4, 5, and 6 was obtained by 677 (4.0%), 2681 (15.7%), 1370 (8.0%), 5264 (30.8%), 570 (3.3%), and 3202 (18.7%) patients, respectively. At this first time point, exceptions for status 1, 2, 3, and 4 were given to 242 (1.4%), 1593 (9.3%), 508 (3.0%), and 1009 (5.9%) patients, respectively.

In status 1, exceptions were notably more Black and Hispanic, less likely to be on ECMO and or invasive mechanical ventilation, and more likely to have an left ventricular assist device (LVAD) (Table 1). Among the hemodynamic criteria, exceptions in status 1 had a slightly higher cardiac index, cardiac output, and systolic blood pressure (Table 2). Status 2 exceptions had lower inotrope and intra-aortic balloon pump (IABP) use, a higher cardiac output, and a lower PCWP than their standard criteria counterparts. In status 3, exceptions were more often female, had a lower body weight and BMI, more likely to have congenital/restrictive cardiomyopathy/hypertrophic cardiomyopathy diagnoses or be retransplants, more frequently listed as heart-kidney, had poorer functional status, and had less LVADs and more inotrope use. They were also more often on vasoactive support and had worse hemodynamic criteria than their standard criteria counterparts. Status 4 exceptions were more often female, older, less likely to have blood type O, had more inotrope use, and almost no LVAD use compared to standard criteria status 4 candidates.

**Table 1:** Demographics at Time of Listing, of all 17,116 Heart Waitlisted Patients, and of Patients With First Active Status 1 to 4, Categorized by Standard Criteria or Exception Listing

Demographic variables	Total (*n* = 17,116)	First active status 1-4 (*n* = 13,344)							
		Std. crit. 1 (*n* = 677)	Exc. 1 (*n* = 242)	Std. crit. 2 (*n* = 2681)	Exc. 2 (*n* = 1593)	Std. crit. 3 (*n* = 1370)	Exc. 3 (*n* = 508)	Std. crit. 4 (*n* = 5264)	Exc. 4 (*n* = 1009)
*At listing*									
Female sex – no. (%)	4532 (26.5)	192 (28.4)	63 (26.0)	639 (23.8)	398 (25.0)	337 (24.6)	165 (32.5)	1398 (26.5)	308 (30.5)
Age (year) – mean ± SD	53.0 ± 12.9	47.7 ± 14.7	49.5 ± 14.4	53.3 ± 13.1	52.1 ± 13.2	52.8 ± 12.7	51.8 ± 13.7	51.6 ± 13.2	55.7 ± 11.5
Weight (kg) – mean ± SD	85.3 ± 18.7	84.9 ± 19.8	85.2 ± 22.2	82.8 ± 18.2	83.8 ± 18.7	86.2 ± 19.8	79.3 ± 18.6	86.9 ± 18.5	84.7 ± 18.0
Body mass index (kg/m^2^) – mean ± SD	28.0 ± 5.0	27.9 ± 5.4	27.6 ± 5.6	27.0 ± 4.9	27.6 ± 5.1	28.2 ± 5.3	26.5 ± 4.9	28.7 ± 4.9	27.8 ± 4.8
Ethnicity									
White – no. (%)	10,003 (58.4)	425 (62.8)	132 (54.6)	1480 (55.2)	853 (53.4)	718 (52.4)	276 (54.3)	3163 (60.1)	611 (60.6)
Black – no. (%)	4483 (26.2)	140 (20.7)	59 (24.4)	742 (27.7)	481 (30.2)	395 (28.8)	136 (26.8)	1387 (26.4)	288 (28.5)
Hispanic – no. (%)	1784 (10.4)	58 (8.6)	32 (13.2)	304 (11.3)	179 (11.2)	170 (12.4)	62 (12.2)	507 (9.6)	76 (7.5)
Other – no. (%)	846 (4.9)	54 (8.0)	19 (7.9)	155 (5.8)	80 (5.0)	87 (6.4)	34 (6.7)	207 (3.9)	34 (3.4)
Blood type									
A – no. (%)	6279 (36.7)	251 (37.1)	79 (32.6)	983 (36.7)	587 (36.9)	521 (38.0)	176 (34.7)	1854 (35.2)	385 (38.2)
B – no. (%)	2522 (14.7)	104 (15.4)	38 (15.7)	428 (16.0)	243 (15.3)	235 (17.2)	82 (16.1)	705 (13.4)	159 (15.8)
AB – no. (%)	771 (4.5)	40 (5.9)	13 (5.4)	104 (3.9)	70 (4.4)	71 (5.2)	31 (6.1)	211 (4.0)	62 (6.1)
O – no. (%)	7544 (44.1)	282 (41.7)	112 (46.3)	1166 (43.5)	693 (43.5)	543 (39.6)	219 (43.1)	2494 (47.4)	403 (39.9)
Diabetes	5203 (30.4)	170 (25.1)	56 (23.1)	786 (29.3)	481 (30.2)	480 (35.0)	143 (28.2)	1536 (29.2)	305 (30.2)
History of smoking	6992 (40.9)	199 (29.4)	80 (33.1)	1021 (38.1)	595 (37.4)	668 (48.8)	162 (31.9)	2215 (42.1)	426 (42.2)
Most recent creatinine^a^ (mg/dl) – median [IQR]	1.2 [1.0-1.5]	1.1 [0.8-1.7]	1.2 [0.8-1.6]	1.2 [0.9-1.6]	1.2 [1.0-1.6]	1.2 [0.9-1.5]	1.2 [1.0-1.7]	1.2 [1.0-1.5]	1.2 [1.0-1.5]
Most recent eGFR^a^ (ml/min/1.73 m^2^) – median [IQR]	62.1 [46.6-80.3]	66.9 [43.6-101.1]	68.7 [48.3-92.0]	64.0 [46.5-83.7]	62.8 [45.6-82.5]	64.6 [49.0-83.0]	59.4 [42.0–80.8]	63.0 [48.4-80.3]	59.1 [46.6-75.9]
Functional status									
Poor – no. (%)	9006 (52.6)	626 (92.5)	213 (88.0)	2249 (83.9)	1331 (83.6)	830 (60.6)	390 (76.8)	1820 (34.6)	403 (39.9)
Moderate – no. (%)	5408 (31.6)	18 (2.7)	15 (6.6)	257 (9.6)	170 (10.7)	348 (25.4)	73 (14.4)	2279 (43.3)	407 (40.3)
Excellent – no. (%)	2016 (11.8)	14 (2.1)	5 (2.1)	79 (3.0)	49 (3.1)	126 (9.2)	22 (4.3)	990 (18.8)	118 (11.7)
Unknown – no. (%)	686 (4.0)	19 (2.8)	8 (3.3)	96 (3.6)	43 (2.7)	66 (4.8)	23 (4.5)	175 (3.3)	81 (8.0)
Inotrope use – no. (%)	5227 (30.5)	291 (43.0)	106 (43.8)	1290 (48.1)	708 (44.4)	526 (38.4)	295 (58.1)	970 (18.4)	567 (56.2)
On ECMO – no. (%)	684 (4.0)	476 (70.3)	131 (54.1)	25 (0.9)	16 (1.0)	8 (0.6)	3 (0.6)	10 (0.2)	1 (0.1)
On ventilator	325 (1.9)	174 (25.7)	47 (19.4)	62 (2.3)	12 (0.8)	2 (0.2)	1 (0.2)	12 (0.2)	3 (0.3)
heart-kidney listing – no. (%)	1986 (11.6)	75 (11.1)	26 (10.7)	299 (11.2)	211 (13.2)	115 (8.4)	99 (19.5)	506 (9.6)	91 (9.0)
Diagnosis									
Ischemic DM – no. (%)	4413 (25.8)	167 (24.7)	49 (20.3)	629 (23.5)	383 (24.0)	375 (27.4)	93 (18.3)	1191 (22.6)	268 (26.6)
Idiopathic DM – no. (%)	5733 (33.5)	184 (27.2)	57 (23.6)	1037 (38.7)	551 (34.6)	549 (40.1)	128 (25.2)	1645 (31.3)	387 (38.4)
Congenital – no. (%)	721 (4.2)	22 (3.3)	10 (4.1)	33 (1.2)	99 (6.2)	14 (1.0)	61 (12.0)	456 (8.7)	3 (0.3)
Retransplant – no. (%)	517 (3.0)	51 (7.5)	21 (8.7)	31 (1.2)	47 (3.0)	5 (0.4)	27 (5.3)	324 (6.2)	0 (0.0)
RCM/HCM – no. (%)	755 (4.4)	15 (2.2)	8 (3.3)	90 (3.4)	95 (6.0)	29 (2.1)	42 (8.3)	340 (6.5)	32 (3.2)
Valvular HD – no. (%)	189 (1.1)	16 (2.4)	4 (1.7)	27 (1.0)	14 (0.9)	11 (0.8)	7 (1.4)	25 (0.5)	10 (1.0)
Other – no. (%)	4788 (28.0)	222 (32.8)	93 (38.4)	834 (31.1)	404 (25.4)	387 (28.3)	150 (29.5)	1283 (24.4)	309 (30.6)
CVA – no. (%)	1299 (7.6)	46 (6.8)	9 (3.7)	189 (7.1)	120 (7.5)	140 (10.2)	35 (6.9)	424 (8.1)	68 (6.7)
AICD – no. (%)	11,438 (66.8)	228 (33.7)	105 (43.4)	1722 (64.2)	972 (61.0)	932 (68.0)	304 (59.8)	3421 (65.0)	827 (82.0)
MCSD									
None – no. (%)	12,478 (72.9)	466 (68.8)	166 (68.6)	2098 (78.3)	1281 (80.4)	606 (44.2)	469 (92.3)	2691 (51.1)	999 (99.0)
LVAD – no. (%)	4384 (25.6)	102 (15.1)	55 (22.7)	492 (18.4)	300 (18.8)	761 (55.6)	38 (7.5)	2564 (48.7)	10 (1.0)
RVAD – no. (%)	36 (0.2)	3 (0.4)	6 (2.5)	14 (0.5)	9 (0.6)	1 (0.1)	1 (0.2)	1 (0.0)	0 (0.0)
TAH – no. (%)	39 (0.2)	0 (0.0)	1 (0.4)	33 (1.2)	0 (0.0)	0 (0.0)	0 (0.0)	2 (0.0)	0 (0.0)
BiVAD – no. (%)	179 (1.1)	106 (15.7)	14 (5.8)	44 (1.6)	3 (0.2)	2 (0.2)	0 (0.0)	6 (0.1)	0 (0.0)
IABP – no. (%)	2357 (13.8)	102 (15.1)	43 (17.8)	1367 (51.0)	612 (38.4)	38 (2.8)	18 (3.5)	74 (1.4)	15 (1.5)

AICD, automatic implantable cardioverter defibrillator; BiVAD, biventricular assist device; CVA, cerebrovascular accident; ECMO, extracorporeal membrane oxygenation; eGFR, estimated glomerular filtration rate; HD, heart disease; IABP, intra-aortic balloon pump; IQR, interquartile range; LVAD, left ventricular assist device; MCSD, mechanical circulatory support device; RCM/HCM, restrictive cardiomyopathy/hypertrophic cardiomyopathy; RVAD, right ventricular assist device; TAH, total artificial heart.

a Medians and interquartile ranges were based on 17,077 patients.

**Table 2:** Risk Stratification Variables in First Active Status 1-4, Categorized by Standard Criteria or Exception Listing

Risk stratification variables		First active status 1–4							
	Availability of data^a^ (*n* = 13,344)	Std. crit. 1	Exc. 1	Std. crit. 2	Exc. 2	Std. crit. 3	Exc. 3	Std. crit. 4	Exc. 4
History of peripheral thromboembolic events – no. (%)	12,361 (94.7)	56 (8.9)	19 (8.2)	225 (8.9)	165 (10.8)	118 (9.2)	30 (6.2)	407 (8.2)	86 (8.8)
On antiarrhythmics – no. (%)	13,314 (99.8)	314 (46.5)	116 (48.5)	1248 (46.8)	805 (50.6)	568 (41.6)	213 (41.9)	2061 (39.2)	492 (48.9)
On dialysis – no. (%)	13,319 (99.8)	90 (13.3)	28 (11.7)	121 (4.5)	82 (5.2)	31 (2.3)	26 (5.1)	83 (1.6)	30 (3.0)
On vasoactive support – no. (%)	13,317 (99.8)	450 (66.8)	153 (64.0)	1909 (71.5)	1044 (65.6)	646 (47.3)	367 (72.2)	1294 (24.6)	766 (76.0)
On invasive mechanical ventilation – no. (%)	13,321 (99.8)	302 (44.7)	76 (31.8)	64 (2.4)	17 (1.1)	1 (0.1)	1 (0.2)	14 (0.3)	0 (0.0)
No. of hospital admissions last year – median [IQR]	11,775 (88.2)	1 [1, 2]	1 [1, 2]	2 [1-3]	2 [1-3]	2 [1-3]	2 [1-3]	1 [0-2]	2 [1, 2]
No. of prior sternotomies – median [IQR]	13,079 (98.0)	0 [0, 1]	0 [0, 1]	0 [0]	0 [0, 1]	1 [0, 1]	0 [0, 1]	1 [0, 1]	0 [0]
Most recent – mean ± SD/median [IQR]									
Cardiac index (liter/min/m^2^)	12,283 (92.0)	2.1 ± 0.9	2.3 ± 0.9	2.1 ± 0.7	2.2 ± 0.7	2.2 ± 0.6	2.3 ± 0.7	2.2 ± 0.7	2.1 ± 0.5
Cardiac output (liter/min)	12,146 (91.0)	4.2 ± 1.8	4.5 ± 1.8	4.1 ± 1.4	4.3 ± 1.4	4.5 ± 1.3	4.3 ± 1.5	4.5 ± 1.2	4.1 ± 1.1
Central venous pressure (mm Hg)	12,134 (90.9)	12.5 ± 6.8	12.5 ± 6.8	10.8 ± 6.3	10.9 ± 6.5	9.1 ± 5.9	10.6 ± 6.4	9.0 ± 5.7	7.8 ± 5.6
Systolic BP (mm Hg)	10,397 (77.9)	95.8 ± 17.3	98.9 ± 19.5	98.2 ± 15.5	104.9 ± 16.8	100.0 ± 15.1	106.4 ± 15.2	107.6 ± 17.2	111.0 ± 17.3
Diastolic BP (mmHg)	10,205 (76.5)	66.0 ± 11.6	67.5 ± 12.4	65.6 ± 11.8	68.0 ± 11.9	69.5 ± 12.8	67.8 ± 11.5	72.8 ± 12.7	70.4 ± 11.4
LVEDP (mm Hg)	40 (0.3)	-	-	23.4 ± 10.6	20.7 ± 7.2	-	18.8 ± 9.9	15.2 ± 7.1	-
PA mean (mm Hg)	12,325 (92.4)	29.0 ± 10.4	30.2 ± 14.7	32.2 ± 9.3	31.3 ± 11.3	26.8 ± 9.5	29.9 ± 10.8	25.2 ± 9.9	26.7 ± 10.2
PA diastolic (mm Hg)	12,526 (93.9)	22.3 ± 8.7	21.8 ± 11.2	23.6 ± 8.0	22.6 ± 9.1	19.1 ± 8.2	21.5 ± 8.5	17.5 ± 8.2	18.4 ± 8.4
PA systolic (mmHg)	12,549 (94.0)	39.3 ± 14.5	43.5 ± 22.1	46.0 ± 12.7	45.6 ± 16.4	39.5 ± 12.8	43.8 ± 16.3	37.7 ± 13.9	39.9 ± 14.4
PCWP (mm Hg)	10,614 (79.5)	23.8 ± 8.9	19.7 ± 9.8	23.2 ± 8.3	21.1 ± 9.2	16.8 ± 8.7	19.1 ± 8.2	16.0 ± 8.2	17.2 ± 8.7
SvO_2_ (%)	9516 (71.3)	58.3 ± 15.9	56.8 ± 13.9	55.5 ± 11.6	56.8 ± 11.1	58.7 ± 10.3	58.3 ± 10.1	62.0 ± 9.0	60.6 ± 9.1
Serum albumin (g/dl)	13,041 (97.7)	3.0 ± 0.6	3.1 ± 0.6	3.5 ± 0.5	3.5 ± 0.6	3.8 ± 0.6	3.7 ± 0.6	4.0 ± 0.6	3.9 ± 0.6
Arterial lactate (mmol/liter)	2987 (22.4)	1.8 ± 2.3	1.7 ± 1.5	1.4 ± 2.0	1.5 ± 2.3	1.7 ± 2.1	1.6 ± 1.8	1.5 ± 2.1	1.3 ± 0.7
AST (U/liter)	13,046 (97.8)	54 [31-123]	43 [26-77]	30 [22-43]	28 [21-40]	25 [19-34]	26 [19-36]	25 [20-33]	24 [19-33]
Serum bilirubin (mg/dl)	13,055 (97.8)	1.3 [0.8-2.2]	0.9 [0.6-1.7]	1 [0.7-1.6]	0.9 [0.6-1.4]	0.7 [0.5-1.1]	0.8 [0.5-1.4]	0.7 [0.5-1]	0.7 [0.5-1]
BNP (pg/ml)	10,914 (81.8)	1917 [779-4673]	2039 [772-4900]	2181 [979-4900]	1525 [567-3804]	841 [277-2445]	1077 [434-2890]	599 [214-1659]	859 [338-2114]
Creatinine (mg/dl)	13,216 (99.0)	1.1 [0.80-1.70]	1.2 [0.8-1.6]	1.2 [0.9-1.6]	1.2 [0.9-1.6]	1.2 [0.9-1.5]	1.25 [0.9-1.7]	1.2 [1-1.5]	1.2 [1-1.5]
INR	12,229 (91.6)	1.3 [1.2-1.6]	1.3 [1.2-1.6]	1.2 [1.1-1.4]	1.2 [1.1-1.4]	1.8 [1.2-2.4]	1.2 [1.1-1.5]	1.8 [1.2-2.4]	1.2 [1-1.7]
Serum sodium (mEq/liter)	13,217 (99.0)	137.6 ± 5.8	136.4 ± 5.4	134.3 ± 4.3	134.7 ± 4.1	136.0 ± 4.0	135.1 ± 4.0	137.6 ± 3.4	137.4 ± 3.6
BUN (mg/dl)	13,212 (99.0)	24 [16-37]	24 [17-38]	22 [16-32]	22 [15-31]	21 [15-28]	23 [17-36]	20 [16-28]	21 [16-29]
Sensitization: CPRA (%)	11,848 (88.8)	8.8 ± 22.2	11.8 ± 25.8	9.3 ± 21.4	10.1 ± 22.4	10.4 ± 22.2	10.3 ± 22.0	13.3 ± 26.4	9.4 ± 22.4
Dobutamine (mcg/kg/min)	2216 (16.6)	4.4 ± 1.9	4.1 ± 2.1	4.2 ± 1.9	4.2 ± 1.8	4.1 ± 1.8	3.9 ± 2.0	4.0 ± 1.5	3.8 ± 1.2
Dopamine (mcg/kg/min)	379 (2.8)	4.1 ± 1.7	3.3 ± 1.6	3.5 ± 1.7	3.6 ± 1.6	3.1 ± 0.7	2.8 ± 1.1	3.1 ± 1.2	3.5 ± 1.3
Ephinephrine (mcg/kg/min)	523 (3.9)	1.3 ± 1.9	0.8 ± 1.7	0.9 ± 1.5	0.7 ± 1.3	0.6 ± 1.2	0.1 ± 0.2	0.9 ± 1.5	-
Norephinephrine (mcg/kg/min)	250 (1.9)	2.2 ± 3.5	1.9 ± 3.9	2.3 ± 3.8	2.0 ± 2.8	8.6 ± 10.7	1.3 ± 1.5	1.1 ± 1.8	-
Milrinone (mcg/kg/min)	4821 (36.1)	0.3 ± 0.1	0.3 ± 0.1	0.3 ± 0.2	0.3 ± 0.1	0.4 ± 0.2	0.4 ± 0.2	0.3 ± 0.1	0.36 ± 0.96
Vasopressin (U/min)	261 (2.0)	0.4 ± 1.2	0.1 ± 0.4	0.3 ± 0.7	0.2 ± 0.4	0.0 ± 0.0	0.0 ± 0.0	1.3 ± 1.4	-

AST, aspartate aminotransferase; BNP, B-type natriuretic peptide; BP, blood pressure; BUN, blood urea nitrogen; CPRA, Calculated Panel Reactive Antibody; INR, international normalized ratio; IQR, interquartile range; LVEDP, left ventricular end diastolic pressure; PA, pulmonary artery; PCWP, pulmonary capillary wedge pressure; SvO_2,_ mixed venous oxygen saturation.

In case of complete data, sample size is n = 677 in standard criteria 1, n = 242 in exception 1, n = 2681 in standard criteria 2, n = 1593 in exception 2, n = 1370 in standard criteria 3, n = 508 in exception 3, n = 5264 in standard criteria 4, n = 1009 in exception 4.

a Not available = not performed or missing.

### Exception usage

**Figure 1:**
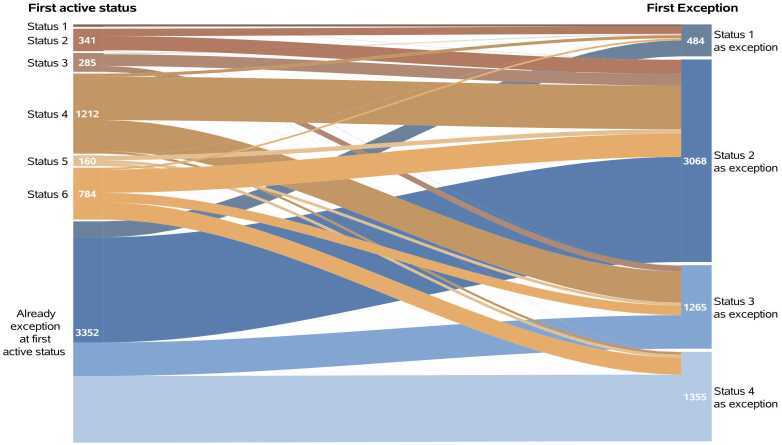
Sankey plot showing the first active status of candidates that were given their first exception (*n* = 6172).

In 54.3% (*n* = 3352) of all patients who were given their first exception, the exception status was the first active status (Figure 1). When this was not the case (*n* = 2820, 45.7%), status 1 exceptions were mainly granted to patients in standard criteria status 2, status 2 exceptions to standard criteria status 4 and 6, status 3 exceptions to standard criteria status 4, and status 4 exceptions to standard criteria status 6. Of all patients in exception status 1 or 2, 91.9% and 82.7%, respectively, remained in that status until either transplantation, death or the end of follow-up. For candidates in exception status 3 or 4, this was only 45.0% and 46.6%, showing that most of these patients changed waitlist status afterward.

### Ranking statuses based on waitlist mortality

**Figure 2:**
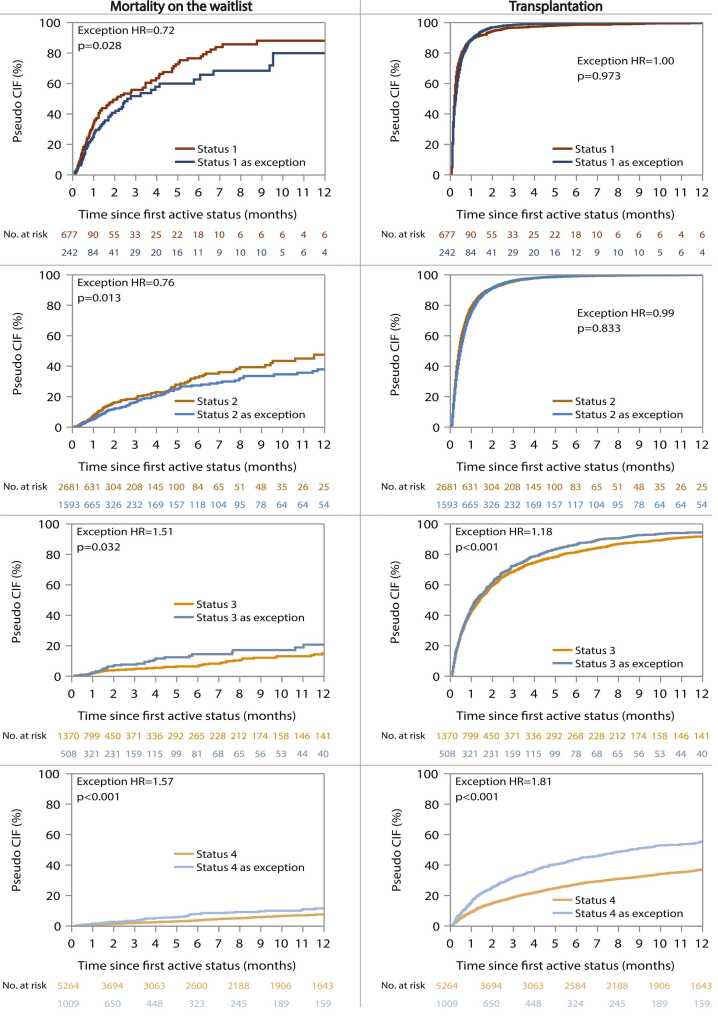
Extended Kaplan-Meier curves for mortality on the waitlist and cardiac transplantation. The reported hazard ratios and *p* values originate from the univariable Cox model, including status as a time-dependent covariate. In extended Kaplan-Meier curves, patients can transition from one status to another during follow-up, as reflected in the sometimes increasing numbers at risk. The curves therefore correspond to hypothetical cohorts of candidates whose waitlist status remained constant throughout follow-up. The cumulative incidence estimates can technically not be interpreted as percentages of a real cohort with events occurring over time.^24^ CIF = cumulative incidence function.

In univariable modeling, compared to status 6 candidates, the overall mortality rate was highest in standard criteria status 1 patients ([hazard ratio] HR 43.47, *p* < 0.001), followed by exception listings in that same status (HR 31.48, *p* < 0.001, Table 3). The mortality rate in status 2 candidates was considerably lower, both for standard criteria listings and for exceptions (HR 10.21, *p* < 0.001 and HR 7.76, *p* < 0.001, respectively). Within these 2 highest priority statuses, exception listings showed consistently lower mortality rates than their standard criteria counterparts (status 1 HR 0.72, *p* = 0.028; status 2 HR 0.76, *p* = 0.013, Table 3 and Figure 2), but they were still much higher compared to the subsequent lower-priority status. The mortality rate of standard criteria status 3 candidates was increased to a lesser extent compared to status 6 (HR 2.61, *p* < 0.001, Table 3), while standard criteria status 4 candidates did not differ significantly from the same reference (HR 1.31, *p* = 0.104). Compared to status 6, exceptions in status 3 and 4 had a hazard ratio of 4.18 (*p* < 0.001) and of 2.13 (*p* < 0.001), respectively. In contrast to the findings in the top 2 statuses, exception listings in status 3 and 4 had an increased waitlist mortality rate compared to their standard criteria counterparts (status 3 HR 1.51, *p* = 0.032; status 4 HR 1.57, *p* < 0.001, Figure 2 and Table 3), indicating a higher urgency for transplant. Apart from the difference between standard criteria status 1 and exceptions in status 1 becoming borderline non-significant (*p* = 0.053), these results were fully corroborated in the multivariable model.

**Table 3:** Univariable and Multivariable Hazard Ratios for the Time to Patient Death on the Heart Transplant Waitlist

Status on the waitlist		Events	Univariable HR (95% CI)	*p* value	Contrasts *p* value	Multivariable HR (95% CI)	*p* value	Contrasts *p* value
Status 1		133	43.47 (33.80-55.90)	<0.001	0.028	52.21 (40.43-67.41)	<0.001	0.053
Status 1 as exception		71	31.48 (23.42-42.31)	<0.001		39.24 (29.09-52.92)	<0.001	
Status 2		191	10.21 (8.08-12.91)	<0.001	0.013	10.48 (8.27-13.27)	<0.001	0.028
Status 2 as exception		147	7.76 (6.08-9.89)	<0.001		8.22 (6.42-10.51)	<0.001	
Status 3		79	2.61 (1.97-3.46)	<0.001	0.032	2.82 (2.12-3.75)	<0.001	0.039
Status 3 as exception		42	3.93 (2.77-5.59)	<0.001		4.19 (2.94-5.97)	<0.001	
Status 4		342	1.31 (1.07-1.61)	0.104	<0.001	1.42 (1.15-1.75)	<0.001	0.009
Status 4 as exception		68	2.05 (1.52-2.76)	<0.001		2.01 (1.49-2.71)	<0.001	
Status 5		71	2.81 (2.10-3.76)	<0.001		2.14 (1.57-2.91)	<0.001	
Status 6		123	1	-		1	-	
*At listing*								
Sex	Female	291	0.96 (0.84-1.10)	0.554		1.03 (0.90-1.18)	0.675	
	Male	976	1	-		1	-	
Race	Black	377	1.11 (0.98-1.25)	0.119		1.02 (0.89-1.16)	0.822	
	Hispanic	126	1.03 (0.85-1.24)	0.799		1.08 (0.89-1.30)	0.459	
	Other	60	1.23 (0.95-1.60)	0.121		0.99 (0.76-1.30)	0.956	
	White	704	1	-		1	-	
Age (per 10 years)		1267	1.20 (1.14-1.26)	<0.001		1.24 (1.18-1.31)	<0.001	
BMI		1267	0.97 (0.96-0.98)	<0.001		0.98 (0.97-0.99)	0.001	
Blood type	A	387	1.05 (0.92-1.19)	0.466		1.15 (1.01-1.31)	0.034	
	B	189	1.31 (1.11-1.54)	0.001		1.23 (1.05-1.46)	0.013	
	AB	39	1.28 (0.93-1.77)	0.136		1.15 (0.83-1.59)	0.413	
	O	652	1	-		1	-	
Diabetes	Yes	442	1.19 (1.06-1.33)	0.003		1.11 (0.98-1.25)	0.104	
	No	825	1	-		1	-	
Smoking history	Yes	538	0.98 (0.87-1.09)	0.669		0.99 (0.89-1.11)	0.924	
	No	729	1	-		1	-	
eGFR (per 10 units)		1265	0.90 (0.89-0.92)	<0.001		0.93 (0.91-0.96)	<0.001	
MCSD	Yes	358	0.74 (0.65-0.83)	<0.001		-	-	
	No	909	1	-		-	-	
Functional status	Excellent	135	0.43 (0.38-0.49)	<0.001		-	-	
	Moderate	367	0.37 (0.31-0.44)	<0.001		-	-	
	Poor	720	1	-		-	-	

BMI, body mass index; CI, confidence interval; eGFR, estimated glomerular filtration rate; HR, hazard ratio; MCSD, mechanical circulatory support device.

The statuses (standard criteria and exceptions) were included as time-varying covariates in the Cox model. Contrasts *p values correspond to the difference between exception and standard criteria listing, within each status (1-4).*

### Ranking statuses based on transplant rate

The transplantation rate did not differ significantly between exception listings and standard criteria listings in status 1 and 2 (status 1, HR 1.00, *p* = 0.973; status 2, HR 0.99, *p* = 0.833) (Figure 2 and Table 4). In contrast, candidates listed in status 3 and 4 via an exception were transplanted significantly sooner than candidates listed via standard criteria (status 3, HR 1.18, *p* < 0.001; status 4, HR 1.81, *p* < 0.001). Compared to status 6, candidates in status 1 (standard criteria HR 29.84, *p* < 0.001; exception HR 29.89, *p* < 0.001) had the highest transplant rate, followed by status 2 (standard criteria HR 21.25, *p* < 0.001; exception HR 21.13, *p* < 0.001), status 3 (standard criteria HR 7.28, *p* < 0.001; exception HR 8.60, *p* < 0.001) and status 4 (standard criteria HR 1.32, *p* < 0.001; exception HR 2.40, *p* < 0.001). These results were confirmed in the multivariable model (Table 4).

**Table 4:** Univariable and Multivariable Hazard Ratios for the Time to Transplant on the Heart Transplant Waitlist

Status on the waitlist		Events	Univariable HR (95% CI)	*p* value	Contrasts *p* value	Multivariable HR (95% CI)	*p* value	Contrasts *p* value
Status 1		772	29.84 (26.85-33.16)	<0.001	0.973	33.42 (30.04-37.18)	<0.001	0.299
Status 1 as exception		499	29.89 (26.56-33.61)	<0.001		35.48 (31.53-39.93)	<0.001	
Status 2		3359	21.25 (19.50-23.14)	<0.001	0.833	23.53 (21.59-25.65)	<0.001	0.266
Status 2 as exception		2891	21.13 (19.39-23.02)	<0.001		24.22 (22.21-26.40)	<0.001	
Status 3		1307	7.28 (6.62-7.99)	<0.001	<0.001	8.21 (7.47-9.02)	<0.001	0.008
Status 3 as exception		599	8.60 (7.69-9.60)	<0.001		9.37 (8.37-10.47)	<0.001	
Status 4		1762	1.32 (1.21-1.45)	<0.001	<0.001	1.48 (1.35-1.62)	<0.001	<0.001
Status 4 as exception		465	2.40 (2.13-2.70)	<0.001		2.46 (2.19-2.77)	<0.001	
Status 5		124	0.91 (0.75-1.12)	0.360		0.99 (0.82-1.21)	0.954	
Status 6		661	1	-		1	-	
*At listing*								
Sex	Female	3391	1.15 (1.10-1.19)	<0.001		1.27 (1.22-1.32)	<0.001	
	Male	9048	1	-		1	-	
Race	Black	3108	0.89 (0.85-0.93)	<0.001		0.77 (0.74-0.81)	<0.001	
	Hispanic	1294	0.99 (0.93-1.05)	0.754		0.98 (0.93-1.05)	0.600	
	Other	647	1.21 (1.11-1.31)	<0.001		0.91 (0.84-0.99)	0.029	
	White	7390	1	-		1	-	
Age (per 10 years)		12,439	1.04 (1.02-1.05)	<0.001		1.12 (1.10-1.13)	<0.001	
BMI		12,439	0.96 (0.96-0.97)	<0.001		0.98 (0.98-0.99)	<0.001	
Blood type	A	4910	1.59 (1.53-1.66)	<0.001		1.78 (1.71-1.85)	<0.001	
	B	1967	1.58 (1.50-1.67)	<0.001		1.58 (1.49-1.66)	<0.001	
	AB	669	2.26 (2.08-2.45)	<0.001		2.46 (2.26-2.66)	<0.001	
	O	4893	1	-		1	-	
Diabetes	Yes	3713	0.95 (0.92-0.99)	0.011		0.98 (0.95-1.03)	0.420	
	No	8726	1	-		1	-	
Smoking history	Yes	4976	0.90 (0.87-0.93)	<0.001		0.96 (0.93-1.00)	0.051	
	No	7463	1	-		1	-	
eGFR (per 10 units)		12,411	1.002 (1.00-1.01)	0.361		1.00 (1.00-1.01)	0.483	
MCSD	Yes	3177	0.72 (0.69-0.75)	<0.001		-	-	
	No	9262	1	-		-	-	
Functional status	Excellent	1181	0.39 (0.37-0.42)	<0.001		-	-	
	Moderate	3624	0.50 (0.48-0.52)	<0.001		-	-	
	Poor	7139	1	-		-	-	

BMI, body mass index; CI, confidence interval; MCSD, mechanical circulatory support device.

The statuses (standard criteria and exceptions) were included as time-varying covariates in the Cox model. Contrasts *p values correspond to the difference between exception and standard criteria listing, within each status (1-4).*

## Discussion

This is a national database study focused on the waitlist mortality of heart candidates listed by standard criteria and exception from November 1, 2018, to December 31, 2022, with follow-up until March 31, 2023. We found that (1) candidates listed by exception in status 1 and 2 had a lower rate of waitlist mortality compared to their standard criteria counterparts, that (2) candidates listed by exception in status 3 and 4 had a higher rate of waitlist mortality compared to their standard criteria counterparts and that (3) exceptions were roughly appropriately placed within in the current 6-tier allocation system according to their mortality rate. We reached the latter conclusion because the mortality rate of exception listings in status 1 and 2 were closest to their standard criteria counterparts and much higher than the lower priority statuses, so there exists no better placement for those exception candidates in the current system. Status 3 and 4 candidates had considerably lower mortality rates compared to status 1 and 2 candidates, and the higher mortality rate for exceptions within these statuses was matched by a higher transplant rate for these same exceptions. Although exceptions in status 3 and 4 had increased waitlist mortality compared to their standard criteria counterparts, mortality rates in these statuses were generally low.

Our findings on status 1 are consistent with the results of Golbus et al and Johnson et al who both reported a lower waitlist mortality rate for exceptions in status 1 compared to standard status 1, while our findings on status 2 clarify their opposing conclusions.^3^, ^5^ Other literature on exception requests have mainly been studied within certain heart failure etiologies, mechanical support devices, and to identify racial inequalities.^9^, ^10^, ^11^, ^12^ We found an increased use of exceptions among candidates with congenital heart disease, restrictive and hypertrophic cardiomyopathy among status 1, 2, and 3 candidates which is consistent with previously reported findings.^9^, ^10^, ^11^

Another notable finding is the use of IABPs at listing for candidates with status 1 and 2 among both standard criteria and exceptions. Candidates with IABP are assigned status 2 for 14 days if they meet certain hemodynamic criteria for cardiogenic shock at listing and can receive an extension for another 14 days if they are unable to be weaned off the IABP and have a contraindication for durable device support.^2^ Candidates with an IABP were likely initially listed as standard criteria status 2 and then moved to status 3 for not meeting the hemodynamic criteria^2^ or received an exception to status 1 or 2. Overall, we observed that candidates with IABPs sometimes petition for and receive even higher priority than the relatively high status afforded by standard policy for candidates with IABPs. A recent OPTN policy proposal aims to modify the IABP eligibility and extension criteria to better align with their waitlist mortality rates.^13^

When the 6-tier allocation system was implemented, UNOS also initiated mandatory data collection on risk stratification variables to facilitate the development of a heart allocation score.^14^, ^15^ The goal was to risk stratify the waitlisted population, based on variables that include data on hemodynamics, heart failure severity and therapies, end organ function, functional status, hospitalizations, and operative risk, so as to lessen the extensive reliance on exceptions to capture mortality risk.^14^, ^15^ Several existing heart failure risk scores that incorporate objective data have been applied to successfully predict heart waitlist mortality in a single-center 4-year cohort.^16^ The 2018 French heart allocation policy^17^ uses the previously reported candidate risk score,^18^ which is comprised of 2 variables indicating end organ function (GFR and Bilirubin) and 2 variables indicating hemodynamic severity of heart failure (ECMO and BNP), to predict the waitlist mortality. They were able to risk stratify the majority of waitlist candidates this way, with points assigned for a small number of candidates needing exceptions where the candidate risk score was not an optimal predictor of mortality. These scores and studies show that risk stratifying patients mainly on objective laboratory criteria might dramatically reduce the need for exception requests.

Our study has some limitations. We were unable to ascertain the reasons for the exception requests, and we did not have information on exception request denials and approvals. We could not account for transplants given to candidates who were eventually denied an exception by the regional review boards. The 4-year heart monitoring report^19^ showed that 88% of status 1% and 86% of status 2 requests for regional review boards approval were exception requests of which 6% of status 1 and 2 requests were denied. With short median days to transplant for status 1 (5 days) and status 2 (13 days) exceptions,^19^ it is likely that these candidates received a heart transplant despite denial, and thereby contributed to the lower mortality rate of exceptions in these high-priority statuses. Next, we did not account for potential behavior changes of transplant staff and regional review board members in requesting and approving exceptions; in February 2021 OPTN issued new guidance regarding how programs should objectively determine their candidates’ medical urgency for requesting a status 2 exception, and how regional review boards should evaluate adult status 2 candidate exception requests.^20^ We also did not account for additional COVID-19 related mortality during the pandemic. Studies showed decreased waitlist mortality^21^ likely due to decreased waitlist additions and increased inactivations^22^ during the pandemic except for those with the highest priority (status 1 and 2). This could have underestimated the overall heart waitlist deaths during the pandemic.

## Conclusions

Exceptions in status 1 and status 2 have lower waitlist mortality than their standard criteria counterparts. However, the hazard of death for status 1 exceptions was significantly higher than that of status 2 standard criteria listings and similarly, status 2 exceptions had a higher hazard of death compared to status 3 standard criteria listings. The Final Rule states that “*rankings shall be ordered from most to least medically urgent” and “there shall be a sufficient number of categories to avoid grouping together patients with substantially different medical urgency*.”^23^ Our study shows that exceptions for status 1 and 2 are appropriately placed in the current allocation system in accordance with the Final Rule. We urge policymakers to adopt a more detailed and precise risk stratification for prioritizing transplants than the current 6-tiered system. A new mortality risk prediction model could be built using the risk stratification variables that have been collected since 2018, and we hope that such a model would capture the waitlist mortality risk for a great number of candidates who in the current system must seek exceptions. The current allocation system requires a large fraction of candidates to seek exceptions, suggesting that we should focus on objective data to risk stratify all heart waitlist candidates, and thereby reduce the need for exceptions.

## Author Contributions

M.C., A.M., and S.G. designed the study and the analysis plan. M.C., A.M., L.J., D.S., N.M., D.S., and S.G. were involved in clinical data collection and data quality control. M.C. did the statistical analyses. M.C. and A.M. created the figures and tables, with input from S.G. M.C., A.M., L.J., D.S., N.M., D.S., and S.G. interpreted the results. M.C., A.M., and S.G. wrote the manuscript, and all coauthors revised and approved it..

## Funding

This work was supported by 10.13039/501100003130Fonds Wetenschappelijk Onderzoek (FWO; Research Foundation – Flanders) [grant number 12D6423N]; and the United States 10.13039/100000002National Institutes of Health (NIH) [grant number K24AI144954]. The funding agencies had no role in the design and conduct of the study; collection, management, analysis, and interpretation of the data; preparation, review, or approval of the manuscript; and decision to submit the manuscript for publication.

## Disclosure statement

The authors declare the following financial interests/personal relationships which may be considered as potential competing interests: Maarten Coemans reports financial support was provided by Research Foundation Flanders. Dorry Segev reports financial support was provided by National Institutes of Health. Dorry Segev reports a relationship with Scientific Registry of Transplant Recipients that includes employment.
